# High Plasma Oxalate Levels Early After Kidney Transplantation Are Associated With Impaired Long-Term Outcomes

**DOI:** 10.3389/ti.2022.10240

**Published:** 2022-03-18

**Authors:** Veronica Krogstad, Katja Benedikte Prestø Elgstøen, Linda Flaa Johnsen, Anders Hartmann, Lars Mørkrid, Anders Åsberg

**Affiliations:** ^1^ Department of Transplantation Medicine, Oslo University Hospital, Oslo, Norway; ^2^ Department of Medical Biochemistry, Oslo University Hospital, Oslo, Norway; ^3^ Institute of Clinical Medicine, Faculty of Medicine, University of Oslo, Oslo, Norway; ^4^ Department of Pharmaceutical Biosciences, School of Pharmacy, University of Oslo, Blindern, Oslo, Norway; ^5^ The Norwegian Renal Registry, Oslo University Hospital, Rikshospitalet, Oslo, Norway

**Keywords:** kidney transplantation, patient survival, graft loss, oxalate, long term outcomes, prospective follow-up

## Abstract

**Background:** Elevated levels of oxalate are common in renal failure patients and non-hyperoxaluria disease, and may cause damage after transplantation. We examined outcomes after 15 years for 167 kidney transplant recipients who had plasma oxalate measured early after transplantation. Analyses included plasma oxalate, recipient age, donor age, live donor, HLA-DR mismatch, mGFR, and smoking.

**Results:** Median age was 52 years (range 18–81), 63% were male and 38% had live donors. Median plasma oxalate concentration 10 weeks after transplantation was 9.0 μmol/L (range 2.7–53.0), one third above the upper reference limit (11.0 μmol/L). Multivariable analysis revealed upper quartile plasma oxalate (>13.0 μmol/L, *p* = 0.008), recipient age (*p* < 0.001), deceased donor (*p* = 0.003), and current smoking (*p* < 0.001) as significant factors associated with patient survival. Upper quartile plasma oxalate (*p* = 0.021), recipient age (*p* = 0.001), deceased donor kidney (*p* = 0.001), HLA-DR mismatch (*p* = 0.015), and current smoking (*p* = 0.014) were also associated with graft loss. Factors associated with death censored graft losses were donor age (*p* = 0.012), deceased donor (*p* = 0.032), and HLA-DR mis-matched kidneys (*p* = 0.005) but plasma oxalate was not (*p* = 0.188).

**Conclusions:** Plasma oxalate in the upper quartile early after transplantation was significantly associated with impaired long-term patient survival and graft losses, but not when censored for death.

## Graphical Abstract

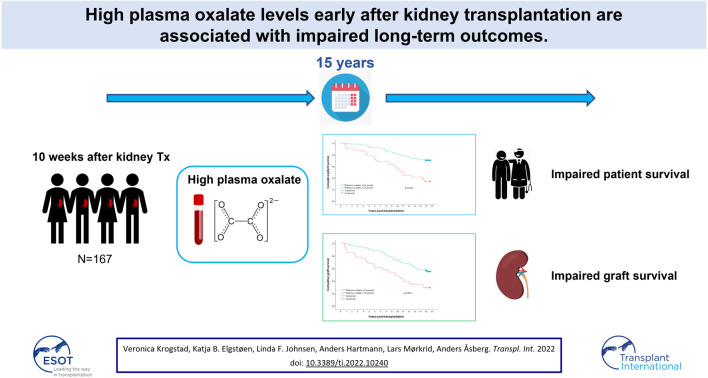

## Introduction

Hyperoxalemia/-oxaluria may cause kidney failure. In typical example cases, it often affects primary hyperoxaluria patients leading to terminal kidney failure at a young age (1). Secondary forms of hyperoxaluria also occur with intestinal disease or following bariatric surgery, which are well recognized to harm the kidneys (2). Another major cause of oxalate retention is kidney failure since the main excretion route for oxalate is glomerular filtration and tubular secretion (3). When patients with kidney failure are successfully treated with a kidney transplant, excess oxalate is excreted by the transplanted kidney and may potentially cause damage.

The retention of oxalate in end-stage renal failure patients without a primary defect in oxalate metabolism has not been well studied. Almost 2 decades ago we started a single center prospective study to assess the outcomes of kidney transplant patients related to levels of plasma oxalate in the perioperative phase (4). We found that more than a third of the patients still had plasma levels of oxalate above the upper reference limit 10 weeks after transplantation and oxalate plasma levels were inversely correlated to kidney graft function. There is growing evidence that oxalate may seriously harm transplanted kidneys (5–7) and possibly also affect mortality (8). The original protocol of our study outlined long-term follow-up of these patients to assess outcomes including patient survival and graft loss. The present study describes the long-term outcomes over 15 years for a cohort of 167 patients who had valid measurements of plasma oxalate 10 weeks after kidney transplantation (4).

## Materials and Methods

### Study Design

In this single-center prospective study, we measured plasma oxalate in kidney transplant recipients in a stable phase, on average 10 weeks after kidney transplantation, consecutively between February 2004 and May 2005. The present study is a long-term follow-up of outcomes in 167 patients that was part of the original protocol, none were lost to follow-up. The design of the single-center prospective study has been previously described in detail (4). Long-term follow-up data on mortality and graft losses were retrieved from the Norwegian Renal Registry until December 2019.

The protocol was approved by the Regional Ethics Committee in South-East Norway and the biobank was approved by the Data Inspectorate. All patients signed informed consent for both the initial study and for biobanking of plasma samples. The study was conducted in accordance with the Declaration of Helsinki.

### Bioanalysis

Plasma oxalate was measured with a validated method as previously described (9). In short, fresh plasma samples were subject to solid-phase extraction followed by derivatization of oxalate and analysis with liquid chromatography-tandem mass spectrometry. All samples were analyzed in duplicate, and the method showed an average CV of 6.9%. Glomerular filtration rate (GFR) at 10 weeks was measured by plasma disappearance of ^51^Cr-EDTA (10). For 10 patients (five patients in the Q1-Q3 group and five patients in the Q4 group), measurement of GFR was not performed, and GFR was estimated for these patients using the MDRD-4 equation (11).

### Statistics

A potential harmful effect of plasma oxalate is only expected at high values. We, therefore, examined the upper quartile versus the other quartiles of plasma oxalate values as predictors for outcomes. The upper quartile had values above 13.0 μmol/L, which is close to the upper reference limit with the present method (11.0 μmol/L).

Kaplan-Meier analyses with a log-rank test were performed to compare patient survival, graft survival, and death-censored graft survival in patients with upper quartile plasma oxalate concentrations (Q4) and the remaining patients (Q1-Q3). Furthermore, univariate and multivariable Cox regression analyses were performed to evaluate the independent effect of post-transplantation plasma oxalate concentration and other clinically relevant risk factors on long-term outcomes. Variables in the univariate analysis with a *p*-value lower than 0.10 for the outcomes and clinically plausible variables were included in the multivariable regression model. Proportional hazards were checked with log-minus-log plots as well as partial residual plots against time rank variables. *p*-values below 0.05 were considered statistically significant. All analyses were performed with SPSS software (IBM, version 26.0.0.1).

## Results

Demographic and transplantation-related baseline data are given for all patients in Table 1, which also includes specified data for the upper quartile of plasma oxalate patients versus the other quartiles of patients combined.

**TABLE 1:** Demographic and transplant data according to quartiles of plasma oxalate. Data presented as median (total range) and number (%).

	All patients (*n* = 167)	Upper quartile (*n* = 41)	Other quartiles (*n* = 126)	*p*
Plasma oxalate 10 weeks after Tx (µmol/L)	9.0 (2.7–53)	16.0 (13.1–53.0)	7.7 (2.7–13.0)	NA
Age (years)	52 (18–81)	59 (22–79)	50 (18–81)	0.002^a^
Male sex	105 (62.9)	30 (73.2)	75 (59.5)	0.116^b^
Preemptive Tx	39 (23.4)	8 (19.5)	31 (24.6)	0.503^b^
Retransplanted patients	27 (16.2)	5 (12.2)	22 (17.5)	0.426^b^
Dialysis time (months)^c^	14 (1–71)	15 (1–71)	12 (1–60)	0.107^a^
Donor beyond 60 years	20 (12.0)	9 (22.0)	11 (8.7)	0.024^b^
Living donor	63 (37.7)	13 (31.7)	50 (39.7)	0.360^b^
HLA-DR mismatch (1 or 2)	108 (64.7)	31 (75.6)	77 (61.1)	0.092^b^
PRA positive	12 (7.2)	3 (7.3)	9 (7.1)	0.970^b^
Cold ischemia time (hours)	7.7 (0.0–24.0)	9.0 (0.8–20.2)	7.0 (0.0–24.0)	0.870^a^
Acute rejection first 10 weeks after Tx	67 (40.1)	19 (46.3)	48 (38.1)	0.349^b^
mGFR 10 weeks after Tx (ml/min)^d^	61 (16–135)	49 (16–90)	64 (30–135)	<0.001^a^
Current smoker	28 (16.8)	8 (19.5)	20 (15.9)	0.588^b^

Abbreviations: HLA-DR, Human Leukocyte Antigen-DR; PRA, Panel Reactive Antibody; Tx, transplantation; mGFR, measured glomerular filtration rate.

a Mann-Whitney *U* test.

b Chi-square test.

c Excluding patients with preemptive transplantation.

d mGFR missing for five patients in the Q1-Q3 group and five patients in the Q4 group. For these patients, eGFR was calculated using the MDRD-4 equation.

The 41 patients in the upper quartile with plasma oxalate values above 13.0 μmol/L had a median plasma oxalate concentration of 16.0 μmol/L, while 126 patients in the other quartiles combined with plasma oxalate concentrations at or below 13.0 μmol/L, had a median plasma oxalate concentration of 7.7 μmol/L.

**FIGURE 1:**
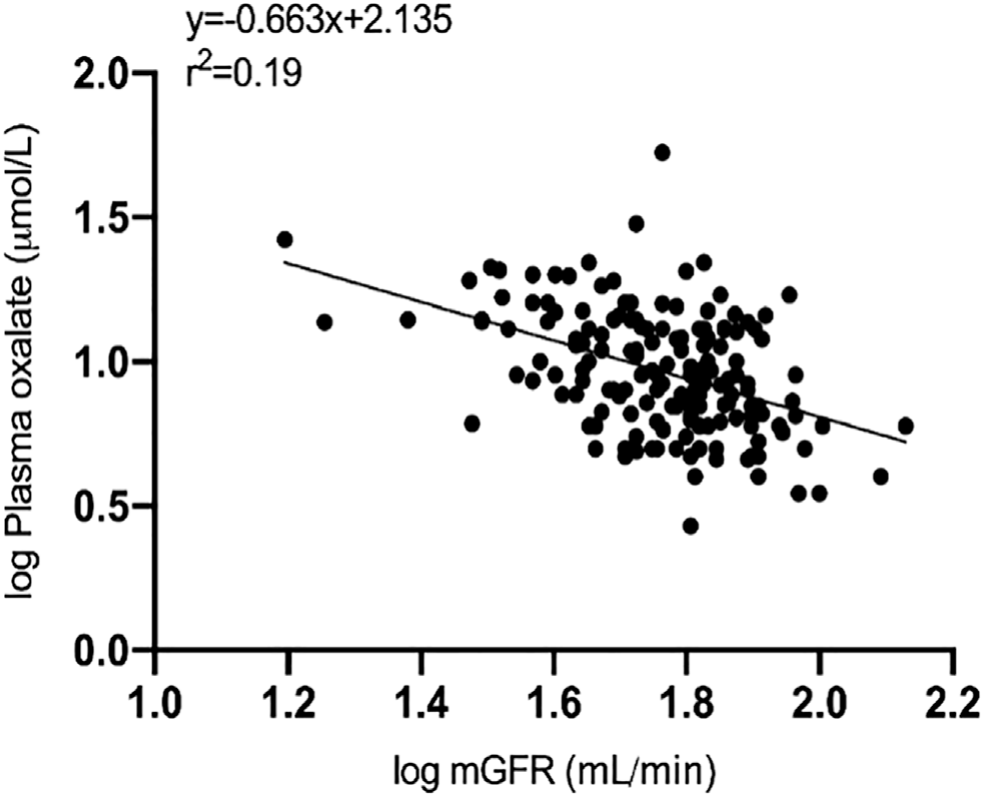
Simple linear regression analysis of logarithmic values of plasma oxalate (µmol/L) as a function of logarithmic values of measured GFR (ml/min) 10 weeks after transplantation (y = −0.663x + 2.135, *r* ^2^ = 0.19, *p* < 0.001).

The upper quartile patients were significantly older and had older donors. They also had substantially lower mGFR compared with the other patients at 10 weeks after transplantation. Plasma oxalate was inversely correlated with mGFR (Figure 1). Transplant demographic data were not different between recipients of a kidney in 2004 and 2005 in which oxalate were measured (*n* = 167) or not (*n* = 326, data not shown).

The median observation time was 15.0 years (range 0.7–15.8). Early rejection episodes were not different between the quartile groups (*p* = 0.35). In the observation period, 64 (38%) patients died and the median time from transplantation to death was 8.2 years (range 0.7–14.8). Uncensored graft loss occurred in 85 patients with a median time from transplantation of 8.2 years (range 0.7–15.1). Death-censored graft loss occurred in 35 (21%) patients, and the median time from transplantation to death-censored graft loss was 9.0 years (range 0.7–15.1).

**FIGURE 2:**
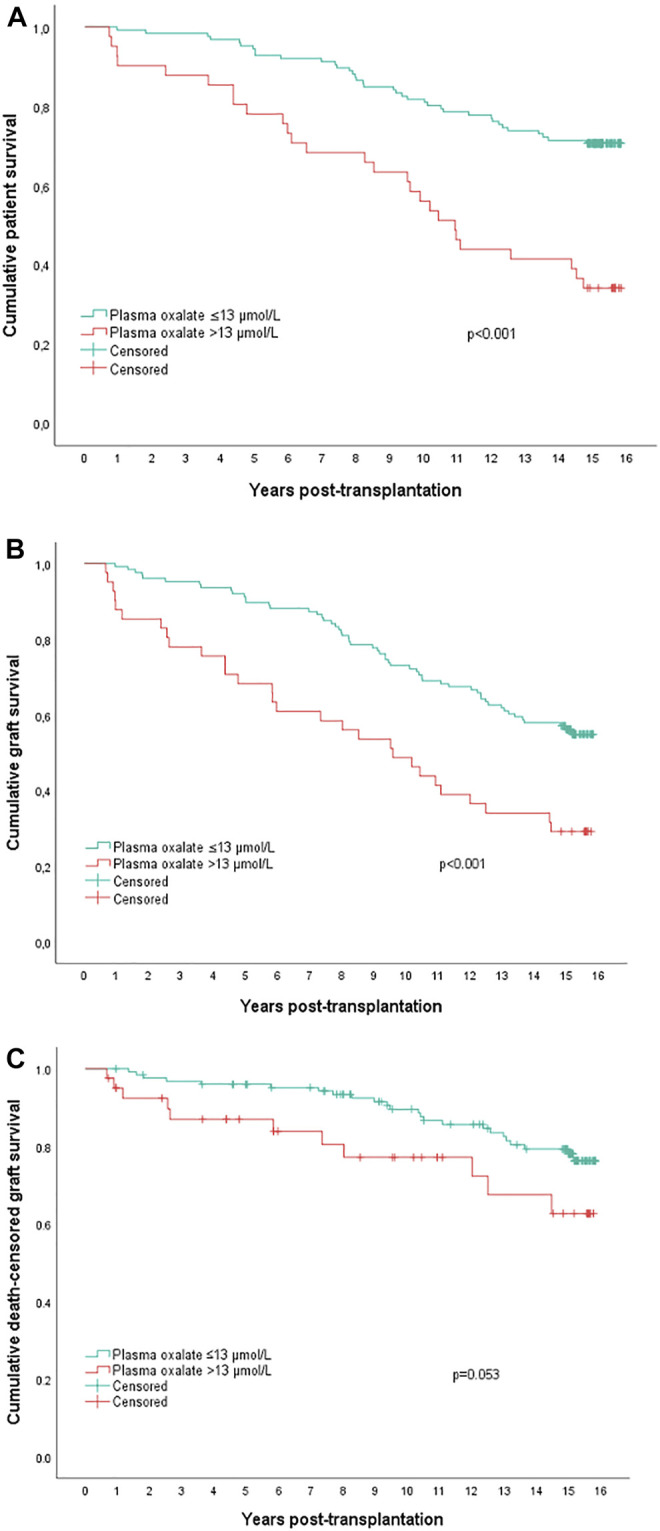
Actuarial **(A)** patient survival, **(B)** overall graft survival, and **(C)** death censored graft survival in kidney transplanted patients with upper quartile plasma oxalate concentrations (>13.0 μmol/L, *n* = 41) versus all other patients (*n* = 126). *p*-values by Log-rank test.

Kaplan-Meier analysis survival plots are shown in Figure 2. The upper panel shows that estimated patient survival was shorter in the upper quartile patients (*p* < 0.0001), with a 15-year survival rate of 34% (95% CI 20–49%) compared with 71% (95% CI 63–79%) for the other patients. Similarly, the uncensored graft survival rate was also shorter in the upper quartile group (*p* < 0.001, middle panel); 15-year graft survival rates of 29% (95% CI 15–43%) and 56% (95% CI 48–64%), respectively. In the lower panel, death-censored graft survival rate is shown, which also tended to be shorter in the upper quartile group (*p* = 0.053). The 15-year death censored graft survival was 63% (95% CI 44–81%) and 78% (95% CI 70–86%) in the respective group.

**FIGURE 3:**
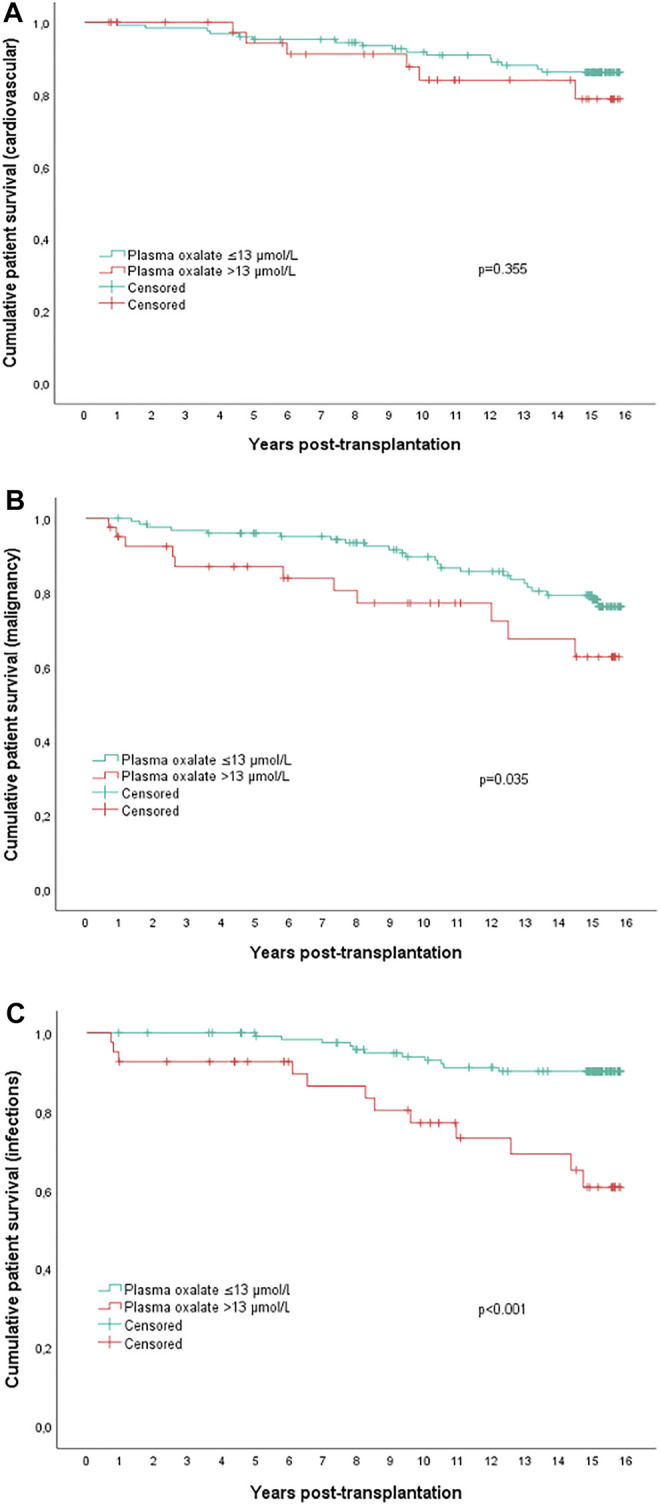
Actuarial **(A)** cardiovascular survival, **(B)** malignancy survival and **(C)** infectious survival in kidney transplanted patients with upper quartile plasma oxalate concentrations (>13.0 μmol/L, *n* = 41) versus all other patients (*n* = 126). *p*-values by Log-rank test.

Kaplan-Meier analyses were also performed to compare mortality due to cardiovascular, malignant, and infectious causes in the upper quartile plasma oxalate patients versus the other patients (Figure 3). Twenty-two patients died from cardiovascular causes; six patients were in the upper quartile group (15%), not significantly different from 16 deaths in the other patient groups combined (13%) (*p* = 0.355). Eleven patients died from malignancy, five patients in the upper quartile group (12%), significantly more than six among the other patients (5%) (*p* = 0.035). Finally, 23 patients died from infectious causes, 12 in the upper quartile group (29%), significantly more than 11 among the other patients (9%) (*p* < 0.001).

Univariate and multivariable Cox regression models for patient survival, graft survival, and death-censored graft survival are shown in Tables 2 and 3. The upper quartile of plasma oxalate along with recipient age, deceased donor kidneys, and current smoking were independently associated with mortality in the multivariable model. Upper quartile plasma oxalate levels, recipient age, deceased donor kidney, and current smoking were also associated with graft loss and in addition also HLA-DR mismatches. The multivariable model for death-censored graft loss revealed donor age over 60 years, deceased donor kidneys, and any HLA-DR mismatch as independent factors. Neither plasma oxalate (*p* = 0.19) nor mGFR (*p* = 0.47) were independently associated with long-term graft loss censored for death. A sensitivity analysis excluding mGFR from the above-mentioned Cox regression showed similar results (Table 4).

**TABLE 2:** Univariate Cox regression analysis of risk factors associated with death, graft loss, or death-censored graft loss.

	Death		Graft loss		Death-censored graft loss	
	HR (95%CI)	*p*	HR (95%CI)	*p*	HR (95%CI)	*p*
Plasma oxalate >13.0 μmol/L after Tx	**3.05 (1.86–5.02)**	**<0.001**	**2.26 (1.44–3.54)**	**<0.001**	2.00 (0.98–4.09)	0.058
Recipient age (years)	**1.09 (1.06–1.11)**	**<0.001**	**1.04 (1.03–1.06)**	**<0.001**	1.00 (0.98–1.02)	0.962
Male sex	1.63 (0.95–2.81)	0.078	1.28 (0.81–2.01)	0.292	1.08 (0.54–2.14)	0.829
Donor > 60 years	1.75 (0.91–3.35)	0.092	**2.03 (1.15–3.61)**	**0.015**	**3.08 (1.40–6.80)**	**0.005**
Living donor	**0.27 (0.14–0.52)**	**<0.001**	**0.42 (0.26–0.68)**	**<0.001**	0.62 (0.31–1.24)	0.176
HLA-DR mismatch (1 or 2)	1.04 (0.62–1.73)	0.882	1.45 (0.91–2.29)	0.117	**3.06 (1.27–7.37)**	**0.013**
Preemptive Tx	0.72 (0.39–1.35)	0.310	0.73 (0.43–1.24)	0.237	0.60 (0.25–1.45)	0.258
PRA positive	1.07 (0.43–2.67)	0.881	1.08 (0.50–2.33)	0.852	0.74 (0.18–3.09)	0.681
mGFR at 10 weeks (ml/min)	**0.98 (0.97–1.00)**	**0.013**	**0.98 (0.97–1.00)**	**0.014**	0.98 (0.96–1.00)	0.117
Current smoker at 10 weeks	1.77 (0.99–3.16)	0.053	1.50 (0.89–2.52)	0.129	0.53 (0.16–1.74)	0.295

Abbreviations: HLA-DR, Human Leukocyte Antigen-DR; PRA, Panel Reactive Antibody; Tx, transplantation; mGFR, measured glomerular filtration rate. Bold data indicate statistical significant findings.

**TABLE 3:** Multivariable Cox regression model of risk factors associated with death, graft loss, or death-censored graft loss.

	Death		Graft loss		Death-censored graft loss	
	HR (95%CI)	*p*	HR (95%CI)	*p*	HR (95%CI)	*p*
Plasma oxalate >13.0 μmol/L	**2.23 (1.24–4.01)**	**0.008**	**1.80 (1.09–2.97)**	**0.021**	1.68 (0.78–3.64)	0.188
Recipient age (years)	**1.08 (1.06–1.11)**	**<0.001**	**1.03 (1.01–1.05)**	**0.001**		
Donor > 60 years	1.13 (0.55–2.32)	0.731	1.50 (0.80–2.81)	0.205	**3.00 (1.27–7.08)**	**0.012**
Living donor	**0.36 (0.18–0.70)**	**0.003**	**0.43 (0.26–0.72)**	**0.001**	**0.45 (0.21–0.93)**	**0.032**
HLA-DR mismatch (1 or 2)	1.10 (0.65–1.85)	0.718	**1.81 (1.12–2.93)**	**0.015**	**3.64 (1.47–9.01)**	**0.005**
mGFR at 10 weeks (ml/min)	1.00 (0.99–1.02)	0.727	0.99 (0.98–1.01)	0.401	0.99 (0.97–1.01)	0.471
Current smoker at 10 weeks	**3.10 (1.69–5.68)**	**<0.001**	**1.96 (1.15–3.35)**	**0.014**	0.67 (0.20–2.25)	0.516

Abbreviations: HLA-DR, Human Leukocyte Antigen-DR; mGFR, measured glomerular filtration rate. Bold data indicate statistical significant findings.

**TABLE 4:** Multivariable Cox regression model of risk factors associated with death, graft loss or death-censored graft loss-excluded mGFR.

	Death		Graft loss		Death-censored graft loss	
	HR (95%CI)	*p*	HR (95%CI)	*p*	HR (95%CI)	*p*
Plasma oxalate >13.0 μmol/L	**2.14 (1.24–3.68)**	0.006	**1.91 (1.18–3.08)**	0.008	1.83 (0.87–3.84)	0.109
Recipient age (years)	**1.08 (1.06–1.11)**	<0.001	**1.03 (1.02–1.05)**	<0.001		
Donor > 60 years	1.10 (0.55–2.18)	0.794	1.60 (0.86–2.95)	0.135	**3.29 (1.44–7.52)**	**0.005**
Living donor	**0.36 (0.19–0.71)**	0.003	**0.43 (0.26–0.71)**	0.001	**0.44 (0.21–0.91)**	**0.027**
HLA-DR mismatch (1 or 2)	1.11 (0.66–1.86)	0.697	**1.76 (1.09–2.82)**	0.020	**3.50 (1.43–8.61)**	**0.006**
Current smoker at 10 weeks	**3.10 (1.69–5.69)**	<0.001	**1.92 (1.12–3.27)**	0.017	0.61 (0.19–2.01)	0.421

Bold data indicate statistical significant findings.

## Discussion

### Patient Survival

The main finding of the present study is that hyperoxalemia in an early stable post-transplant phase is associated with impaired long-term survival for 15 years. This is a novel finding. A previous retrospective study of 67 patients with calcium oxalate deposits in biopsies taken early after transplantation showed that such deposits were associated with impaired outcomes after 5 years (8). The outcome was a composite end-point of death and graft loss but graft loss was a major contributor to the combined end-point. They did not address mortality *per se*. In fact, in our study, we also found a significant effect of hyperoxalemia on uncensored graft losses, i.e., the combination of deaths and graft losses.

One might question the reason for the association between hyperoxalemia and impaired long-term patient survival as demonstrated in the present study. The patients with hyperoxalemia in the upper quartile were older, more of them also had donors beyond 60 years and their graft function was significantly lower at baseline, i.e., 10 weeks after transplantation. These are well-acknowledged risk factors for patient and graft survival. Nevertheless, in the multivariable analysis including these covariates, the effect of hyperoxalemia on mortality remained strong. The risk of dying was twice as high for the patients with hyperoxalemia in the upper quartile. Due to covariation between renal function and plasma oxalate concentrations the inclusion of both mGFR and plasma oxalate in the same multivariable Cox-analysis may be questioned. However, the results outlined above also hold true when mGFR is left out of the analysis (Table 4). Although the most common cause of death was cardiovascular, we did not find a significant effect of hyperoxalemia on cardiovascular mortality. On the other hand, the effect on malignancy deaths and particularly infectious deaths were markedly increased. These associations are hard to explain. The number of events is limited in these analyses, and one may only speculate whether hyperoxalemia has any causal relation to the cause of death. However, in kidney transplanted patients in general there is an increased risk for both malignancies and infectious deaths due to obligatory immunosuppressive therapy and also due to previous long-term kidney failure.

The immunosuppressive regimen during follow-up after transplantation is standardized on a national level and should not be different between the upper quartile plasma oxalate patients and the other patients. Rejection episodes are treated with steroids and often the immunosuppressive regimen is strengthened, but there was no significant difference in rejection episodes between the groups that could explain the different infectious death outcomes.

In a recent study, the effect of hyperoxaluria on mortality was addressed in a cohort of stable transplanted patients more than a year after transplantation (12). During 7 years of follow-up there was a significant reduction in mortality among patients with hyperoxaluria, mainly driven by a reduction of infectious disease related deaths. We did not measure urinary excretion but addressed plasma levels of oxalate in an early phase after transplantation that may be more relevant to early harmful effects. In any case, the reason for the difference in outcomes between the present study and the study by Tubben et al. (12) remains speculative.

### Graft Survival

As mentioned above we found an association of hyperoxalemia and graft loss when including mortality, but when patients who died with functioning grafts were censored, the association was no longer significant. The only significant factors for such an association were HLA-DR mismatch, deceased donor kidney, and high donor age, as would be expected. The lack of associations to graft outcomes in the present study may be surprising since numerous other publications have shown kidney damage related to hyperoxaluria and calciumoxalate deposits (2,5-7) In the kidney, oxalate microcrystals may cause programmed inflammation and necrosis and also mitochondrial damage leading to necrosis in distal tubular cells and acute kidney injury (13,14). Hyperoxaluria in kidney transplant patients in the study from Tubben et. al. did not reveal any effect on graft loss during 7 years follow-up (12). However, a much larger study in more than 3,000 chronic kidney disease patients with similar observation time found that hyperoxaluria was associated with a 30% increase in the progression of kidney disease and also end-stage kidney disease (15). There may be differences between these patients and kidney transplanted patients but the kidney function was similar in the studies.

Oxalate deposits are shown to have an impact on kidney graft outcomes. One biopsy study examined renal outcomes in 67 patients who had oxalate deposits in early biopsies with 70 control patients. Those who had deposits in the biopsies had worse kidney function at one, but not at 2 years, but with significantly more interstitial scarring than controls (6). Also two other biopsy studies revealed that calcium oxalate deposits showed association to impaired graft function and long-term graft loss during up to 12 years (5,6). We did not examine oxalate deposits in biopsies but oxalate deposits are associated with high plasma levels, leading to increased filtration and probably also secretion of oxalate in a functioning kidney, leading to hyperoxaluria (3).

The number of death censored graft losses in the present study was only 35, limiting the possibility to reveal any association to hyperoxalemia.

The strengths of the present analysis are the prospective design with long-term outcomes included in the original protocol. The cohort of transplant patients was unselected, and none of them was lost to follow-up. It was, however, a weakness that no urine samples were obtained for oxalate data, and no biopsies were obtained for oxalate deposition or nephrocalcinosis. The time course of the covariates after 10 weeks might also be an unknown modifying factor.

*In conclusion*, plasma oxalate concentration in the upper quartile early after transplantation is significantly associated with impaired long-term patient and graft survival but not when graft losses were censored for death with functioning grafts.

## Data Availability

The data underlying this article will be shared on reasonable request to the corresponding author. A specific ethical approval may be needed before data sharing is possible according to Norwegian law.
